# Crystal structure of μ-oxido-1,1′κ^2^ *O*:*O*-bis{tetra-μ-oxido-1:2κ^2^ *O*:*O*;1:3κ^2^ *O*:*O*;2:3κ^4^ *O*:*O*-tris[1,2,3(η^5^)-penta­methyl­cyclo­penta­dien­yl]-*trianglo*-trititanium(IV)}

**DOI:** 10.1107/S2056989015004041

**Published:** 2015-03-21

**Authors:** Adrián Pérez-Redondo, Avelino Martín

**Affiliations:** a Química Inorgánica, Universidad de Alcalá, Campus Universitario, ES 28871 Alcalá de Henares (Madrid), Spain

**Keywords:** crystal structure, titanium oxide, penta­methyl­cyclo­penta­dienyl ligand, organometallic

## Abstract

The title polynuclear organometallic titanium(IV) oxide, [{Ti_3_(η^5^-C_5_Me_5_)_3_(μ-O)_4_}_2_(μ-O)], exhibits two Ti_3_O_4_ cores bridged by an O atom located on a twofold axis. All metal centres present the typical three-legged piano-stool coordination environment, where one site is occupied by a penta­methyl­cyclo­penta­dienyl ligand linked in an η^5^-coordination fashion, while three bridging O atoms fill the other three sites.

## Related literature   

For comparison Ti—O bond lengths in other reported organometallic titanium(IV) complexes, see: Andrés *et al.* (1996 ▸); Carofiglio *et al.* (1992 ▸); Gómez-Sal *et al.* (1996 ▸). For the structures of related titanium derivatives, see: Carbó *et al.* (2009 ▸).
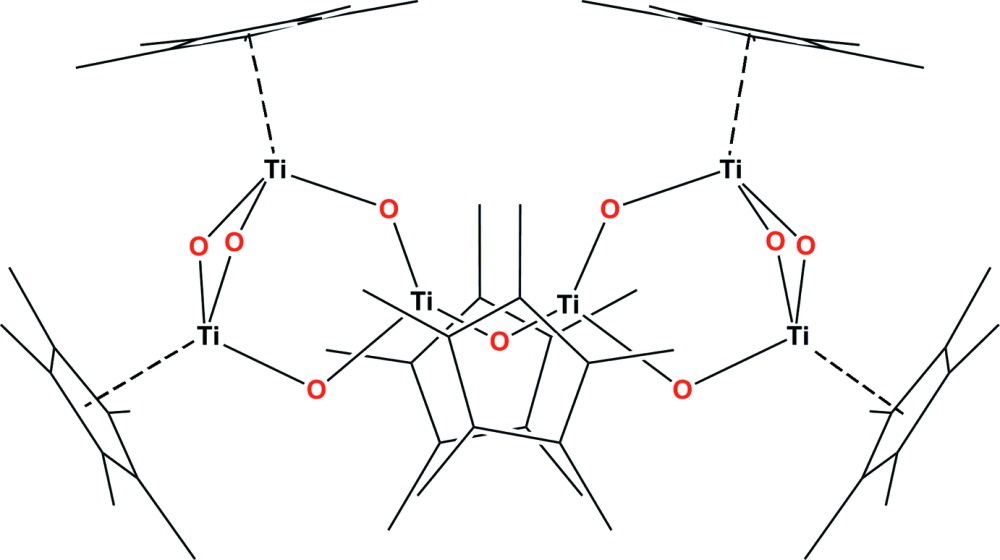


## Experimental   

### Crystal data  

[Ti_6_O_9_(C_10_H_15_)_6_]
*M* *_r_* = 1242.71
Monoclinic, 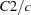

*a* = 31.086 (6) Å
*b* = 11.414 (4) Å
*c* = 17.900 (5) Å
β = 101.265 (8)°
*V* = 6229 (3) Å^3^
*Z* = 4
Mo *K*α radiation
μ = 0.78 mm^−1^
*T* = 200 K
0.44 × 0.24 × 0.15 mm

### Data collection  

Nonius KappaCCD diffractometer
Absorption correction: multi-scan (Blessing, 1995 ▸) *T*_min_ = 0.791, *T*_max_ = 0.864
64311 measured reflections
7159 independent reflections
5059 reflections with *I* > 2σ(*I*)
*R*_int_ = 0.121

### Refinement  

*R*[*F* ^2^ > 2σ(*F* ^2^)] = 0.056
*wR*(*F* ^2^) = 0.133
*S* = 1.14
7159 reflections
354 parameters
H-atom parameters constrained
Δρ_max_ = 0.40 e Å^−3^
Δρ_min_ = −0.42 e Å^−3^

### 

Data collection: *COLLECT* (Nonius, 1998 ▸); cell refinement: *DIRAX/LSQ* (Duisenberg *et al.*, 2000 ▸); data reduction: *EVALCCD* (Duisenberg *et al.*, 2003 ▸); program(s) used to solve structure: SHELXT-2014 (Sheldrick, 2015*a* ▸); program(s) used to refine structure: *SHELXL2014*/7 (Sheldrick, 2015*b* ▸); molecular graphics: *DIAMOND* (Brandenburg, 2006 ▸); software used to prepare material for publication: *WinGX* (Farrugia, 2012 ▸) and *publCIF* (Westrip, 2010 ▸).

## Supplementary Material

Crystal structure: contains datablock(s) I. DOI: 10.1107/S2056989015004041/rz5150sup1.cif

Structure factors: contains datablock(s) I. DOI: 10.1107/S2056989015004041/rz5150Isup2.hkl

Supporting information file. DOI: 10.1107/S2056989015004041/rz5150Isup3.mol

**Fig. 1.**
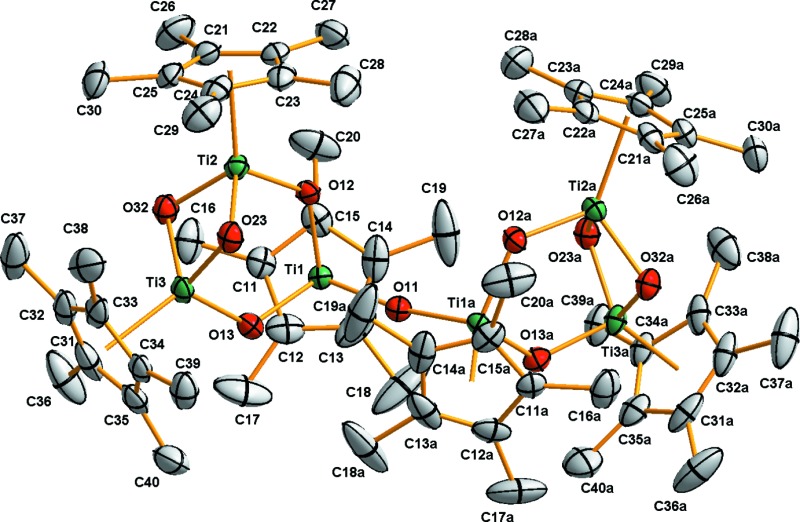
The molecular structure of the title compound with displacement ellipsoids drawn at the 50% of probability level. Hydrogen atoms are omitted for clarity. Atoms labelled with suffix a are generated by the symmetry operation (-x + 1, y, -z + 1/2).

Click here for additional data file.x y z . DOI: 10.1107/S2056989015004041/rz5150fig1.tif
The mol­ecular structure of the title compound with displacement ellipsoids drawn at the 50% of probability level. Hydrogen atoms are omitted for clarity. Atoms labelled with suffix a are generated by the symmetry operation (−*x* + 1, *y*, −*z* + 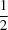).

CCDC reference: 1051365

Additional supporting information:  crystallographic information; 3D view; checkCIF report

## supplementary crystallographic information

### S1. Structural commentary

The averaged Ti—O bond distance of 1.857 (3) Å is similar to that found in other reported organometallic titanium(IV) complexes (Andrés *et al.*, 1996; Carofiglio *et al.*, 1992; Gómez-Sal *et al.*, 1996). On the other hand, the presence of a second bridging oxygen atom between two titanium atoms leads to different Ti···Ti distances in the core (Ti2···Ti3 = 2.6977 (12) Å; mean value of 3.300 (9) Å for Ti1···Ti2 and Ti1···Ti3). This shorter distance has also been found in several methyl­idene or ethyl­idene titanium derivatives (Carbó *et al.*, 2009).

### S2. Refinement

All H atoms were placed geometrically and refined using a rotating-group model, with C–H = 0.98 Å, and with *U*_iso_(H) = 1.5*U*_eq_(C).

### Crystal data

**Table:**

[Ti_6_O_9_(C_10_H_15_)_6_]	*F*(000) = 2616
*M**_r_* = 1242.71	*D*_x_ = 1.325 Mg m^−^^3^
Monoclinic, *C*2/*c*	Mo *K*α radiation, λ = 0.71073 Å
Hall symbol: -C 2yc	Cell parameters from 169 reflections
*a* = 31.086 (6) Å	θ = 3–20°
*b* = 11.414 (4) Å	µ = 0.78 mm^−^^1^
*c* = 17.900 (5) Å	*T* = 200 K
β = 101.265 (8)°	Prism, yellow
*V* = 6229 (3) Å^3^	0.44 × 0.24 × 0.15 mm
*Z* = 4	

### Data collection

**Table:**

Nonius KappaCCD diffractometer	7159 independent reflections
Radiation source: Enraf Nonius FR590	5059 reflections with *I* > 2σ(*I*)
Horizonally mounted graphite crystal monochromator	*R*_int_ = 0.121
Detector resolution: 9 pixels mm^-1^	θ_max_ = 27.5°, θ_min_ = 3.1°
CCD rotation images, thick slices scans	*h* = −40→40
Absorption correction: multi-scan (Blessing, 1995)	*k* = −14→14
*T*_min_ = 0.791, *T*_max_ = 0.864	*l* = −23→23
64311 measured reflections	

### Refinement

**Table:**

Refinement on *F*^2^	Primary atom site location: other
Least-squares matrix: full	Secondary atom site location: difference Fourier map
*R*[*F*^2^ > 2σ(*F*^2^)] = 0.056	Hydrogen site location: inferred from neighbouring sites
*wR*(*F*^2^) = 0.133	H-atom parameters constrained
*S* = 1.14	*w* = 1/[σ^2^(*F*_o_^2^) + (0.0399*P*)^2^ + 19.8847*P*] where *P* = (*F*_o_^2^ + 2*F*_c_^2^)/3
7159 reflections	(Δ/σ)_max_ = 0.001
354 parameters	Δρ_max_ = 0.40 e Å^−^^3^
0 restraints	Δρ_min_ = −0.42 e Å^−^^3^
0 constraints	

### Special details

**Table:**

Geometry. All e.s.d.'s (except the e.s.d. in the dihedral angle between two l.s. planes) are estimated using the full covariance matrix. The cell e.s.d.'s are taken into account individually in the estimation of e.s.d.'s in distances, angles and torsion angles; correlations between e.s.d.'s in cell parameters are only used when they are defined by crystal symmetry. An approximate (isotropic) treatment of cell e.s.d.'s is used for estimating e.s.d.'s involving l.s. planes.

### Fractional atomic coordinates and isotropic or equivalent isotropic displacement parameters (Å^2^)

**Table:**

	*x*	*y*	*z*	*U*_iso_*/*U*_eq_	
C11	0.56708 (12)	0.1643 (3)	0.46931 (19)	0.0303 (8)	
C12	0.54916 (15)	0.0603 (3)	0.4323 (2)	0.0404 (10)	
C13	0.50441 (15)	0.0807 (4)	0.4002 (2)	0.0494 (12)	
C14	0.49427 (12)	0.1986 (4)	0.4170 (2)	0.0439 (11)	
C15	0.53317 (12)	0.2494 (3)	0.45971 (19)	0.0337 (8)	
C16	0.61296 (14)	0.1811 (5)	0.5147 (2)	0.0514 (12)	
H16A	0.6131	0.1672	0.5688	0.077*	
H16B	0.6227	0.2614	0.5078	0.077*	
H16C	0.6329	0.1257	0.497	0.077*	
C17	0.5736 (2)	−0.0537 (4)	0.4302 (3)	0.0803 (19)	
H17A	0.5602	−0.1145	0.4568	0.12*	
H17B	0.6043	−0.0432	0.4553	0.12*	
H17C	0.5722	−0.0774	0.3771	0.12*	
C18	0.4727 (2)	−0.0087 (6)	0.3574 (3)	0.102 (3)	
H18A	0.4886	−0.0618	0.3293	0.153*	
H18B	0.4496	0.0318	0.3216	0.153*	
H18C	0.4595	−0.0538	0.3937	0.153*	
C19	0.45057 (15)	0.2594 (7)	0.3929 (3)	0.083 (2)	
H19A	0.4344	0.2235	0.346	0.124*	
H19B	0.4554	0.3426	0.3839	0.124*	
H19C	0.4335	0.2515	0.4333	0.124*	
C20	0.5369 (2)	0.3712 (4)	0.4916 (3)	0.0630 (15)	
H20A	0.5305	0.3703	0.5431	0.094*	
H20B	0.516	0.4225	0.459	0.094*	
H20C	0.5668	0.4005	0.4936	0.094*	
C21	0.63834 (12)	0.5896 (3)	0.4018 (2)	0.0350 (8)	
C22	0.59681 (12)	0.6137 (3)	0.3540 (2)	0.0361 (9)	
C23	0.60229 (13)	0.6103 (3)	0.2776 (2)	0.0384 (9)	
C24	0.64753 (13)	0.5865 (3)	0.2766 (2)	0.0354 (8)	
C25	0.66974 (12)	0.5733 (3)	0.3536 (2)	0.0329 (8)	
C26	0.64853 (17)	0.5837 (5)	0.4873 (3)	0.0583 (13)	
H26A	0.6211	0.586	0.5067	0.087*	
H26B	0.6669	0.6506	0.5076	0.087*	
H26C	0.6642	0.5107	0.5036	0.087*	
C27	0.55380 (15)	0.6360 (4)	0.3793 (3)	0.0577 (13)	
H27A	0.5397	0.7057	0.3534	0.087*	
H27B	0.5594	0.6485	0.4345	0.087*	
H27C	0.5345	0.5681	0.3664	0.087*	
C28	0.56669 (16)	0.6293 (5)	0.2082 (3)	0.0657 (15)	
H28A	0.5435	0.5707	0.2073	0.099*	
H28B	0.5791	0.6215	0.1621	0.099*	
H28C	0.5543	0.708	0.21	0.099*	
C29	0.66696 (16)	0.5788 (4)	0.2056 (3)	0.0518 (11)	
H29A	0.6984	0.5623	0.22	0.078*	
H29B	0.6626	0.6534	0.1781	0.078*	
H29C	0.6525	0.5158	0.1729	0.078*	
C30	0.71762 (13)	0.5490 (4)	0.3818 (3)	0.0503 (11)	
H30A	0.7321	0.5394	0.3383	0.075*	
H30B	0.721	0.4771	0.4123	0.075*	
H30C	0.731	0.6147	0.4133	0.075*	
C31	0.70057 (14)	0.0474 (4)	0.3430 (2)	0.0494 (12)	
C32	0.72430 (13)	0.1512 (4)	0.3340 (2)	0.0452 (11)	
C33	0.71384 (11)	0.1844 (4)	0.2563 (2)	0.0389 (9)	
C34	0.68353 (11)	0.0992 (3)	0.2165 (2)	0.0326 (8)	
C35	0.67556 (13)	0.0139 (4)	0.2702 (2)	0.0394 (9)	
C36	0.7033 (2)	−0.0215 (6)	0.4163 (3)	0.081 (2)	
H36A	0.7297	−0.0704	0.4248	0.121*	
H36B	0.6773	−0.0714	0.4124	0.121*	
H36C	0.7047	0.033	0.459	0.121*	
C37	0.75520 (15)	0.2143 (6)	0.3969 (3)	0.0768 (19)	
H37A	0.7687	0.2806	0.3754	0.115*	
H37B	0.7781	0.1601	0.4214	0.115*	
H37C	0.7388	0.2429	0.4348	0.115*	
C38	0.73128 (15)	0.2888 (4)	0.2202 (3)	0.0585 (13)	
H38A	0.74	0.3498	0.2587	0.088*	
H38B	0.7084	0.3195	0.1793	0.088*	
H38C	0.7568	0.2652	0.1991	0.088*	
C39	0.66364 (13)	0.1013 (4)	0.1325 (2)	0.0457 (10)	
H39A	0.6834	0.0618	0.1041	0.069*	
H39B	0.6594	0.1827	0.1153	0.069*	
H39C	0.6353	0.0609	0.1237	0.069*	
C40	0.64629 (16)	−0.0915 (4)	0.2541 (3)	0.0575 (13)	
H40A	0.6182	−0.0751	0.2691	0.086*	
H40B	0.6604	−0.1586	0.2831	0.086*	
H40C	0.6412	−0.1095	0.1995	0.086*	
O11	0.5	0.1924 (3)	0.25	0.0244 (7)	
O12	0.56240 (7)	0.36493 (19)	0.33456 (13)	0.0262 (5)	
O13	0.59427 (8)	0.1294 (2)	0.30996 (14)	0.0293 (5)	
O23	0.62819 (8)	0.3292 (2)	0.24333 (14)	0.0314 (6)	
O32	0.65416 (8)	0.3079 (2)	0.38476 (14)	0.0318 (6)	
Ti1	0.54534 (2)	0.20794 (5)	0.33346 (3)	0.02093 (14)	
Ti2	0.61744 (2)	0.41678 (5)	0.32569 (4)	0.02450 (15)	
Ti3	0.64719 (2)	0.19959 (6)	0.30476 (4)	0.02685 (15)	

### Atomic displacement parameters (Å^2^)

**Table:**

	*U*^11^	*U*^22^	*U*^33^	*U*^12^	*U*^13^	*U*^23^
C11	0.036 (2)	0.0352 (19)	0.0194 (17)	0.0001 (16)	0.0051 (14)	0.0080 (14)
C12	0.062 (3)	0.031 (2)	0.029 (2)	−0.0032 (18)	0.0127 (19)	0.0095 (16)
C13	0.059 (3)	0.065 (3)	0.024 (2)	−0.035 (2)	0.0072 (19)	0.008 (2)
C14	0.0267 (19)	0.085 (3)	0.0226 (18)	0.000 (2)	0.0102 (15)	0.015 (2)
C15	0.040 (2)	0.045 (2)	0.0186 (17)	0.0089 (17)	0.0107 (15)	0.0055 (15)
C16	0.041 (2)	0.078 (3)	0.031 (2)	0.000 (2)	−0.0023 (18)	0.016 (2)
C17	0.149 (6)	0.033 (2)	0.062 (3)	0.017 (3)	0.028 (4)	0.016 (2)
C18	0.131 (6)	0.124 (5)	0.042 (3)	−0.101 (5)	−0.004 (3)	0.017 (3)
C19	0.037 (3)	0.170 (7)	0.044 (3)	0.032 (3)	0.017 (2)	0.034 (3)
C20	0.111 (4)	0.050 (3)	0.034 (2)	0.021 (3)	0.028 (3)	−0.002 (2)
C21	0.035 (2)	0.0296 (19)	0.041 (2)	−0.0095 (16)	0.0080 (16)	−0.0090 (16)
C22	0.0305 (19)	0.0209 (17)	0.056 (3)	−0.0055 (15)	0.0063 (18)	−0.0043 (17)
C23	0.035 (2)	0.0265 (19)	0.050 (2)	−0.0057 (16)	0.0001 (18)	0.0073 (17)
C24	0.038 (2)	0.0302 (19)	0.037 (2)	−0.0105 (16)	0.0046 (16)	0.0065 (16)
C25	0.0257 (18)	0.0306 (19)	0.041 (2)	−0.0091 (15)	0.0027 (15)	−0.0018 (16)
C26	0.065 (3)	0.065 (3)	0.044 (3)	−0.014 (3)	0.006 (2)	−0.019 (2)
C27	0.043 (3)	0.038 (2)	0.097 (4)	0.002 (2)	0.025 (3)	−0.013 (2)
C28	0.054 (3)	0.055 (3)	0.076 (4)	−0.005 (2)	−0.016 (3)	0.021 (3)
C29	0.057 (3)	0.054 (3)	0.047 (3)	−0.016 (2)	0.017 (2)	0.007 (2)
C30	0.028 (2)	0.059 (3)	0.061 (3)	−0.0122 (19)	−0.0003 (19)	0.003 (2)
C31	0.042 (2)	0.066 (3)	0.039 (2)	0.034 (2)	0.0050 (19)	0.003 (2)
C32	0.0261 (19)	0.068 (3)	0.038 (2)	0.015 (2)	−0.0003 (17)	−0.020 (2)
C33	0.0196 (17)	0.054 (2)	0.045 (2)	0.0038 (16)	0.0098 (16)	−0.0155 (19)
C34	0.0233 (17)	0.041 (2)	0.033 (2)	0.0105 (15)	0.0049 (14)	−0.0087 (16)
C35	0.035 (2)	0.040 (2)	0.044 (2)	0.0181 (17)	0.0100 (18)	−0.0045 (18)
C36	0.087 (4)	0.106 (5)	0.051 (3)	0.057 (4)	0.018 (3)	0.031 (3)
C37	0.039 (3)	0.129 (5)	0.056 (3)	0.018 (3)	−0.009 (2)	−0.046 (3)
C38	0.042 (2)	0.057 (3)	0.085 (4)	−0.008 (2)	0.034 (3)	−0.019 (3)
C39	0.034 (2)	0.067 (3)	0.036 (2)	0.007 (2)	0.0064 (17)	−0.009 (2)
C40	0.058 (3)	0.036 (2)	0.081 (4)	0.011 (2)	0.018 (3)	−0.005 (2)
O11	0.0256 (17)	0.0259 (17)	0.0223 (16)	0	0.0061 (13)	0
O12	0.0233 (12)	0.0245 (12)	0.0330 (13)	−0.0002 (9)	0.0108 (10)	−0.0041 (10)
O13	0.0286 (13)	0.0262 (12)	0.0333 (14)	0.0043 (10)	0.0068 (10)	−0.0011 (10)
O23	0.0273 (13)	0.0398 (14)	0.0279 (13)	−0.0008 (11)	0.0076 (10)	−0.0008 (11)
O32	0.0281 (13)	0.0389 (14)	0.0274 (13)	0.0018 (11)	0.0029 (10)	−0.0025 (11)
Ti1	0.0217 (3)	0.0216 (3)	0.0202 (3)	−0.0007 (2)	0.0059 (2)	0.0005 (2)
Ti2	0.0225 (3)	0.0249 (3)	0.0268 (3)	−0.0029 (2)	0.0064 (2)	−0.0009 (3)
Ti3	0.0206 (3)	0.0333 (3)	0.0268 (3)	0.0050 (3)	0.0052 (2)	−0.0040 (3)

### Geometric parameters (Å, º)

**Table:**

C11—C15	1.418 (5)	C28—H28B	0.98
C11—C12	1.420 (5)	C28—H28C	0.98
C11—C16	1.510 (5)	C29—H29A	0.98
C11—Ti1	2.444 (3)	C29—H29B	0.98
C12—C13	1.416 (6)	C29—H29C	0.98
C12—C17	1.511 (6)	C30—H30A	0.98
C12—Ti1	2.429 (4)	C30—H30B	0.98
C13—C14	1.427 (7)	C30—H30C	0.98
C13—C18	1.517 (6)	C31—C32	1.422 (7)
C13—Ti1	2.398 (4)	C31—C35	1.433 (6)
C14—C15	1.422 (6)	C31—C36	1.517 (7)
C14—C19	1.512 (6)	C31—Ti3	2.408 (4)
C14—Ti1	2.386 (4)	C32—C33	1.418 (6)
C15—C20	1.499 (6)	C32—C37	1.512 (6)
C15—Ti1	2.410 (4)	C32—Ti3	2.415 (4)
C16—H16A	0.98	C33—C34	1.441 (5)
C16—H16B	0.98	C33—C38	1.506 (6)
C16—H16C	0.98	C33—Ti3	2.404 (4)
C17—H17A	0.98	C34—C35	1.424 (6)
C17—H17B	0.98	C34—C39	1.510 (5)
C17—H17C	0.98	C34—Ti3	2.404 (3)
C18—H18A	0.98	C35—C40	1.502 (6)
C18—H18B	0.98	C35—Ti3	2.422 (4)
C18—H18C	0.98	C36—H36A	0.98
C19—H19A	0.98	C36—H36B	0.98
C19—H19B	0.98	C36—H36C	0.98
C19—H19C	0.98	C37—H37A	0.98
C20—H20A	0.98	C37—H37B	0.98
C20—H20B	0.98	C37—H37C	0.98
C20—H20C	0.98	C38—H38A	0.98
C21—C22	1.429 (5)	C38—H38B	0.98
C21—C25	1.435 (5)	C38—H38C	0.98
C21—C26	1.504 (6)	C39—H39A	0.98
C21—Ti2	2.413 (4)	C39—H39B	0.98
C22—C23	1.410 (6)	C39—H39C	0.98
C22—C27	1.515 (6)	C40—H40A	0.98
C22—Ti2	2.418 (4)	C40—H40B	0.98
C23—C24	1.436 (5)	C40—H40C	0.98
C23—C28	1.510 (6)	O11—Ti1	1.8513 (7)
C23—Ti2	2.383 (4)	O11—Ti1^i^	1.8513 (7)
C24—C25	1.424 (5)	O12—Ti2	1.846 (2)
C24—C29	1.512 (6)	O12—Ti1	1.868 (2)
C24—Ti2	2.392 (4)	O13—Ti3	1.849 (2)
C25—C30	1.501 (5)	O13—Ti1	1.883 (2)
C25—Ti2	2.401 (3)	O23—Ti2	1.865 (3)
C26—H26A	0.98	O23—Ti3	1.869 (3)
C26—H26B	0.98	O32—Ti2	1.869 (3)
C26—H26C	0.98	O32—Ti3	1.872 (3)
C27—H27A	0.98	Ti1—Ti2	3.2934 (11)
C27—H27B	0.98	Ti1—Ti3	3.3065 (10)
C27—H27C	0.98	Ti2—Ti3	2.6977 (12)
C28—H28A	0.98		
			
C15—C11—C12	107.5 (3)	H37A—C37—H37C	109.5
C15—C11—C16	125.5 (4)	H37B—C37—H37C	109.5
C12—C11—C16	126.9 (4)	C33—C38—H38A	109.5
C15—C11—Ti1	71.68 (19)	C33—C38—H38B	109.5
C12—C11—Ti1	72.5 (2)	H38A—C38—H38B	109.5
C16—C11—Ti1	123.9 (2)	C33—C38—H38C	109.5
C13—C12—C11	108.4 (4)	H38A—C38—H38C	109.5
C13—C12—C17	126.2 (4)	H38B—C38—H38C	109.5
C11—C12—C17	125.3 (4)	C34—C39—H39A	109.5
C13—C12—Ti1	71.7 (2)	C34—C39—H39B	109.5
C11—C12—Ti1	73.7 (2)	H39A—C39—H39B	109.5
C17—C12—Ti1	122.2 (3)	C34—C39—H39C	109.5
C12—C13—C14	108.0 (4)	H39A—C39—H39C	109.5
C12—C13—C18	125.7 (5)	H39B—C39—H39C	109.5
C14—C13—C18	126.3 (5)	C35—C40—H40A	109.5
C12—C13—Ti1	74.1 (2)	C35—C40—H40B	109.5
C14—C13—Ti1	72.2 (2)	H40A—C40—H40B	109.5
C18—C13—Ti1	120.7 (3)	C35—C40—H40C	109.5
C15—C14—C13	107.3 (4)	H40A—C40—H40C	109.5
C15—C14—C19	126.1 (5)	H40B—C40—H40C	109.5
C13—C14—C19	126.6 (5)	Ti1—O11—Ti1^i^	169.0 (2)
C15—C14—Ti1	73.7 (2)	Ti2—O12—Ti1	124.93 (12)
C13—C14—Ti1	73.1 (2)	Ti3—O13—Ti1	124.80 (13)
C19—C14—Ti1	118.1 (3)	Ti2—O23—Ti3	92.52 (11)
C11—C15—C14	108.7 (4)	Ti2—O32—Ti3	92.28 (11)
C11—C15—C20	126.0 (4)	O11—Ti1—O12	105.68 (12)
C14—C15—C20	125.3 (4)	O11—Ti1—O13	107.16 (9)
C11—C15—Ti1	74.4 (2)	O12—Ti1—O13	102.66 (10)
C14—C15—Ti1	71.9 (2)	O11—Ti1—C14	90.37 (10)
C20—C15—Ti1	121.6 (3)	O12—Ti1—C14	104.99 (14)
C11—C16—H16A	109.5	O13—Ti1—C14	141.63 (14)
C11—C16—H16B	109.5	O11—Ti1—C13	87.12 (12)
H16A—C16—H16B	109.5	O12—Ti1—C13	138.85 (14)
C11—C16—H16C	109.5	O13—Ti1—C13	110.70 (15)
H16A—C16—H16C	109.5	C14—Ti1—C13	34.70 (16)
H16B—C16—H16C	109.5	O11—Ti1—C15	122.77 (10)
C12—C17—H17A	109.5	O12—Ti1—C15	84.15 (12)
C12—C17—H17B	109.5	O13—Ti1—C15	125.78 (12)
H17A—C17—H17B	109.5	C14—Ti1—C15	34.49 (14)
C12—C17—H17C	109.5	C13—Ti1—C15	57.02 (15)
H17A—C17—H17C	109.5	O11—Ti1—C12	116.25 (13)
H17B—C17—H17C	109.5	O12—Ti1—C12	133.20 (13)
C13—C18—H18A	109.5	O13—Ti1—C12	84.59 (13)
C13—C18—H18B	109.5	C14—Ti1—C12	57.08 (15)
H18A—C18—H18B	109.5	C13—Ti1—C12	34.12 (15)
C13—C18—H18C	109.5	C15—Ti1—C12	56.46 (13)
H18A—C18—H18C	109.5	O11—Ti1—C11	143.34 (10)
H18B—C18—H18C	109.5	O12—Ti1—C11	99.33 (12)
C14—C19—H19A	109.5	O13—Ti1—C11	92.69 (12)
C14—C19—H19B	109.5	C14—Ti1—C11	57.05 (13)
H19A—C19—H19B	109.5	C13—Ti1—C11	56.73 (13)
C14—C19—H19C	109.5	C15—Ti1—C11	33.96 (12)
H19A—C19—H19C	109.5	C12—Ti1—C11	33.87 (13)
H19B—C19—H19C	109.5	O11—Ti1—Ti2	116.89 (8)
C15—C20—H20A	109.5	O12—Ti1—Ti2	27.36 (7)
C15—C20—H20B	109.5	O13—Ti1—Ti2	75.47 (8)
H20A—C20—H20B	109.5	C14—Ti1—Ti2	126.90 (12)
C15—C20—H20C	109.5	C13—Ti1—Ti2	153.01 (10)
H20A—C20—H20C	109.5	C15—Ti1—Ti2	97.49 (10)
H20B—C20—H20C	109.5	C12—Ti1—Ti2	126.59 (11)
C22—C21—C25	107.8 (3)	C11—Ti1—Ti2	97.54 (9)
C22—C21—C26	127.2 (4)	O11—Ti1—Ti3	118.23 (3)
C25—C21—C26	125.0 (4)	O12—Ti1—Ti3	75.57 (7)
C22—C21—Ti2	73.0 (2)	O13—Ti1—Ti3	27.33 (7)
C25—C21—Ti2	72.2 (2)	C14—Ti1—Ti3	150.52 (9)
C26—C21—Ti2	121.2 (3)	C13—Ti1—Ti3	132.64 (13)
C23—C22—C21	108.0 (3)	C15—Ti1—Ti3	118.81 (10)
C23—C22—C27	125.1 (4)	C12—Ti1—Ti3	100.44 (11)
C21—C22—C27	126.9 (4)	C11—Ti1—Ti3	93.54 (9)
C23—C22—Ti2	71.6 (2)	Ti2—Ti1—Ti3	48.25 (2)
C21—C22—Ti2	72.6 (2)	O12—Ti2—O23	102.03 (11)
C27—C22—Ti2	120.3 (3)	O12—Ti2—O32	102.24 (11)
C22—C23—C24	108.7 (3)	O23—Ti2—O32	84.66 (11)
C22—C23—C28	125.9 (4)	O12—Ti2—C23	101.95 (12)
C24—C23—C28	125.4 (4)	O23—Ti2—C23	105.33 (13)
C22—C23—Ti2	74.3 (2)	O32—Ti2—C23	151.16 (12)
C24—C23—Ti2	72.8 (2)	O12—Ti2—C24	136.61 (12)
C28—C23—Ti2	119.5 (3)	O23—Ti2—C24	90.02 (13)
C25—C24—C23	107.4 (3)	O32—Ti2—C24	120.45 (12)
C25—C24—C29	127.5 (4)	C23—Ti2—C24	34.99 (13)
C23—C24—C29	125.1 (4)	O12—Ti2—C25	145.26 (12)
C25—C24—Ti2	73.1 (2)	O23—Ti2—C25	110.15 (12)
C23—C24—Ti2	72.2 (2)	O32—Ti2—C25	93.61 (12)
C29—C24—Ti2	120.7 (3)	C23—Ti2—C25	57.58 (13)
C24—C25—C21	108.0 (3)	C24—Ti2—C25	34.56 (13)
C24—C25—C30	127.4 (4)	O12—Ti2—C21	111.44 (12)
C21—C25—C30	124.6 (4)	O23—Ti2—C21	144.52 (12)
C24—C25—Ti2	72.4 (2)	O32—Ti2—C21	99.03 (13)
C21—C25—Ti2	73.1 (2)	C23—Ti2—C21	57.23 (14)
C30—C25—Ti2	121.3 (3)	C24—Ti2—C21	57.56 (13)
C21—C26—H26A	109.5	C25—Ti2—C21	34.69 (13)
C21—C26—H26B	109.5	O12—Ti2—C22	89.55 (11)
H26A—C26—H26B	109.5	O23—Ti2—C22	139.42 (13)
C21—C26—H26C	109.5	O32—Ti2—C22	131.09 (13)
H26A—C26—H26C	109.5	C23—Ti2—C22	34.15 (14)
H26B—C26—H26C	109.5	C24—Ti2—C22	57.49 (14)
C22—C27—H27A	109.5	C25—Ti2—C22	57.41 (12)
C22—C27—H27B	109.5	C21—Ti2—C22	34.42 (13)
H27A—C27—H27B	109.5	O12—Ti2—Ti3	93.78 (8)
C22—C27—H27C	109.5	O23—Ti2—Ti3	43.81 (8)
H27A—C27—H27C	109.5	O32—Ti2—Ti3	43.90 (8)
H27B—C27—H27C	109.5	C23—Ti2—Ti3	148.23 (11)
C23—C28—H28A	109.5	C24—Ti2—Ti3	121.60 (10)
C23—C28—H28B	109.5	C25—Ti2—Ti3	118.42 (10)
H28A—C28—H28B	109.5	C21—Ti2—Ti3	140.16 (10)
C23—C28—H28C	109.5	C22—Ti2—Ti3	174.57 (10)
H28A—C28—H28C	109.5	O12—Ti2—Ti1	27.71 (7)
H28B—C28—H28C	109.5	O23—Ti2—Ti1	82.76 (8)
C24—C29—H29A	109.5	O32—Ti2—Ti1	80.81 (8)
C24—C29—H29B	109.5	C23—Ti2—Ti1	126.72 (10)
H29A—C29—H29B	109.5	C24—Ti2—Ti1	156.92 (10)
C24—C29—H29C	109.5	C25—Ti2—Ti1	165.54 (10)
H29A—C29—H29C	109.5	C21—Ti2—Ti1	132.72 (9)
H29B—C29—H29C	109.5	C22—Ti2—Ti1	116.98 (9)
C25—C30—H30A	109.5	Ti3—Ti2—Ti1	66.13 (3)
C25—C30—H30B	109.5	O13—Ti3—O23	100.99 (11)
H30A—C30—H30B	109.5	O13—Ti3—O32	102.64 (11)
C25—C30—H30C	109.5	O23—Ti3—O32	84.45 (11)
H30A—C30—H30C	109.5	O13—Ti3—C33	145.61 (13)
H30B—C30—H30C	109.5	O23—Ti3—C33	92.31 (13)
C32—C31—C35	108.7 (4)	O32—Ti3—C33	110.17 (12)
C32—C31—C36	125.9 (5)	O13—Ti3—C34	111.32 (12)
C35—C31—C36	125.2 (5)	O23—Ti3—C34	97.23 (12)
C32—C31—Ti3	73.2 (2)	O32—Ti3—C34	144.91 (12)
C35—C31—Ti3	73.3 (2)	C33—Ti3—C34	34.88 (13)
C36—C31—Ti3	122.6 (3)	O13—Ti3—C31	104.10 (15)
C33—C32—C31	108.0 (4)	O23—Ti3—C31	149.27 (14)
C33—C32—C37	126.5 (5)	O32—Ti3—C31	106.78 (14)
C31—C32—C37	125.5 (5)	C33—Ti3—C31	57.03 (16)
C33—C32—Ti3	72.5 (2)	C34—Ti3—C31	57.09 (14)
C31—C32—Ti3	72.6 (2)	O13—Ti3—C32	138.18 (15)
C37—C32—Ti3	120.8 (3)	O23—Ti3—C32	119.66 (15)
C32—C33—C34	107.9 (4)	O32—Ti3—C32	91.35 (12)
C32—C33—C38	126.8 (4)	C33—Ti3—C32	34.21 (14)
C34—C33—C38	125.3 (4)	C34—Ti3—C32	57.30 (13)
C32—C33—Ti3	73.3 (2)	C31—Ti3—C32	34.29 (16)
C34—C33—Ti3	72.6 (2)	O13—Ti3—C35	90.31 (13)
C38—C33—Ti3	120.4 (3)	O23—Ti3—C35	129.17 (13)
C35—C34—C33	108.2 (3)	O32—Ti3—C35	141.24 (13)
C35—C34—C39	126.3 (4)	C33—Ti3—C35	57.46 (14)
C33—C34—C39	125.6 (4)	C34—Ti3—C35	34.31 (13)
C35—C34—Ti3	73.5 (2)	C31—Ti3—C35	34.51 (14)
C33—C34—Ti3	72.6 (2)	C32—Ti3—C35	57.31 (15)
C39—C34—Ti3	119.5 (2)	O13—Ti3—Ti2	93.36 (8)
C34—C35—C31	107.2 (4)	O23—Ti3—Ti2	43.67 (8)
C34—C35—C40	126.7 (4)	O32—Ti3—Ti2	43.82 (8)
C31—C35—C40	126.1 (4)	C33—Ti3—Ti2	117.36 (11)
C34—C35—Ti3	72.2 (2)	C34—Ti3—Ti2	138.33 (10)
C31—C35—Ti3	72.2 (2)	C31—Ti3—Ti2	149.28 (12)
C40—C35—Ti3	120.9 (3)	C32—Ti3—Ti2	122.19 (12)
C31—C36—H36A	109.5	C35—Ti3—Ti2	172.55 (10)
C31—C36—H36B	109.5	O13—Ti3—Ti1	27.87 (7)
H36A—C36—H36B	109.5	O23—Ti3—Ti1	82.32 (8)
C31—C36—H36C	109.5	O32—Ti3—Ti1	80.40 (8)
H36A—C36—H36C	109.5	C33—Ti3—Ti1	167.73 (10)
H36B—C36—H36C	109.5	C34—Ti3—Ti1	134.66 (9)
C32—C37—H37A	109.5	C31—Ti3—Ti1	127.18 (13)
C32—C37—H37B	109.5	C32—Ti3—Ti1	155.88 (12)
H37A—C37—H37B	109.5	C35—Ti3—Ti1	118.18 (10)
C32—C37—H37C	109.5	Ti2—Ti3—Ti1	65.62 (2)
			
C15—C11—C12—C13	−0.2 (4)	C38—C33—C34—C35	179.3 (3)
C16—C11—C12—C13	176.6 (4)	Ti3—C33—C34—C35	−65.4 (2)
Ti1—C11—C12—C13	−63.8 (3)	C32—C33—C34—C39	179.6 (3)
C15—C11—C12—C17	−178.3 (4)	C38—C33—C34—C39	−1.0 (6)
C16—C11—C12—C17	−1.5 (6)	Ti3—C33—C34—C39	114.2 (3)
Ti1—C11—C12—C17	118.2 (4)	C32—C33—C34—Ti3	65.4 (3)
C15—C11—C12—Ti1	63.6 (2)	C38—C33—C34—Ti3	−115.3 (4)
C16—C11—C12—Ti1	−119.7 (4)	C33—C34—C35—C31	0.6 (4)
C11—C12—C13—C14	0.3 (4)	C39—C34—C35—C31	−179.0 (3)
C17—C12—C13—C14	178.3 (4)	Ti3—C34—C35—C31	−64.1 (3)
Ti1—C12—C13—C14	−64.7 (3)	C33—C34—C35—C40	−179.6 (4)
C11—C12—C13—C18	−178.3 (4)	C39—C34—C35—C40	0.7 (6)
C17—C12—C13—C18	−0.3 (7)	Ti3—C34—C35—C40	115.6 (4)
Ti1—C12—C13—C18	116.7 (4)	C33—C34—C35—Ti3	64.8 (2)
C11—C12—C13—Ti1	65.0 (3)	C39—C34—C35—Ti3	−114.9 (4)
C17—C12—C13—Ti1	−117.0 (4)	C32—C31—C35—C34	−1.0 (4)
C12—C13—C14—C15	−0.2 (4)	C36—C31—C35—C34	−177.4 (4)
C18—C13—C14—C15	178.3 (4)	Ti3—C31—C35—C34	64.1 (2)
Ti1—C13—C14—C15	−66.2 (2)	C32—C31—C35—C40	179.3 (4)
C12—C13—C14—C19	178.9 (4)	C36—C31—C35—C40	2.8 (6)
C18—C13—C14—C19	−2.6 (6)	Ti3—C31—C35—C40	−115.6 (4)
Ti1—C13—C14—C19	112.9 (4)	C32—C31—C35—Ti3	−65.1 (3)
C12—C13—C14—Ti1	66.0 (3)	C36—C31—C35—Ti3	118.5 (4)
C18—C13—C14—Ti1	−115.5 (4)	Ti1^i^—O11—Ti1—O12	−13.07 (7)
C12—C11—C15—C14	0.1 (4)	Ti1^i^—O11—Ti1—O13	−122.02 (8)
C16—C11—C15—C14	−176.8 (3)	Ti1^i^—O11—Ti1—C14	92.63 (12)
Ti1—C11—C15—C14	64.2 (2)	Ti1^i^—O11—Ti1—C13	127.19 (13)
C12—C11—C15—C20	177.9 (4)	Ti1^i^—O11—Ti1—C15	80.08 (13)
C16—C11—C15—C20	1.1 (6)	Ti1^i^—O11—Ti1—C12	145.55 (12)
Ti1—C11—C15—C20	−118.0 (4)	Ti1^i^—O11—Ti1—C11	118.16 (19)
C12—C11—C15—Ti1	−64.1 (2)	Ti1^i^—O11—Ti1—Ti2	−39.94 (4)
C16—C11—C15—Ti1	119.1 (3)	Ti1^i^—O11—Ti1—Ti3	−94.84 (6)
C13—C14—C15—C11	0.1 (4)	Ti2—O12—Ti1—O11	−118.68 (14)
C19—C14—C15—C11	−179.0 (4)	Ti2—O12—Ti1—O13	−6.52 (18)
Ti1—C14—C15—C11	−65.8 (2)	Ti2—O12—Ti1—C14	146.61 (16)
C13—C14—C15—C20	−177.8 (4)	Ti2—O12—Ti1—C13	137.3 (2)
C19—C14—C15—C20	3.1 (6)	Ti2—O12—Ti1—C15	118.88 (17)
Ti1—C14—C15—C20	116.4 (4)	Ti2—O12—Ti1—C12	88.0 (2)
C13—C14—C15—Ti1	65.9 (2)	Ti2—O12—Ti1—C11	88.39 (17)
C19—C14—C15—Ti1	−113.2 (4)	Ti2—O12—Ti1—Ti3	−2.89 (13)
C25—C21—C22—C23	1.0 (4)	Ti3—O13—Ti1—O11	118.74 (16)
C26—C21—C22—C23	−179.9 (4)	Ti3—O13—Ti1—O12	7.69 (18)
Ti2—C21—C22—C23	−63.2 (3)	Ti3—O13—Ti1—C14	−127.6 (2)
C25—C21—C22—C27	179.4 (4)	Ti3—O13—Ti1—C13	−147.80 (16)
C26—C21—C22—C27	−1.5 (6)	Ti3—O13—Ti1—C15	−84.2 (2)
Ti2—C21—C22—C27	115.1 (4)	Ti3—O13—Ti1—C12	−125.43 (18)
C25—C21—C22—Ti2	64.3 (3)	Ti3—O13—Ti1—C11	−92.50 (17)
C26—C21—C22—Ti2	−116.6 (4)	Ti3—O13—Ti1—Ti2	4.60 (13)
C21—C22—C23—C24	−1.3 (4)	Ti1—O12—Ti2—O23	46.93 (18)
C27—C22—C23—C24	−179.7 (4)	Ti1—O12—Ti2—O32	−40.13 (18)
Ti2—C22—C23—C24	−65.2 (3)	Ti1—O12—Ti2—C23	155.68 (17)
C21—C22—C23—C28	179.2 (4)	Ti1—O12—Ti2—C24	149.98 (17)
C27—C22—C23—C28	0.8 (6)	Ti1—O12—Ti2—C25	−155.35 (18)
Ti2—C22—C23—C28	115.3 (4)	Ti1—O12—Ti2—C21	−145.07 (16)
C21—C22—C23—Ti2	63.9 (3)	Ti1—O12—Ti2—C22	−172.27 (18)
C27—C22—C23—Ti2	−114.5 (4)	Ti1—O12—Ti2—Ti3	3.44 (15)
C22—C23—C24—C25	1.0 (4)	Ti3—O23—Ti2—O12	−82.84 (11)
C28—C23—C24—C25	−179.4 (4)	Ti3—O23—Ti2—O32	18.57 (11)
Ti2—C23—C24—C25	−65.1 (3)	Ti3—O23—Ti2—C23	171.02 (11)
C22—C23—C24—C29	−178.4 (4)	Ti3—O23—Ti2—C24	139.17 (12)
C28—C23—C24—C29	1.1 (6)	Ti3—O23—Ti2—C25	110.47 (12)
Ti2—C23—C24—C29	115.5 (4)	Ti3—O23—Ti2—C21	116.6 (2)
C22—C23—C24—Ti2	66.1 (3)	Ti3—O23—Ti2—C22	173.49 (14)
C28—C23—C24—Ti2	−114.3 (4)	Ti3—O23—Ti2—Ti1	−62.81 (8)
C23—C24—C25—C21	−0.4 (4)	Ti3—O32—Ti2—O12	82.64 (11)
C29—C24—C25—C21	179.0 (4)	Ti3—O32—Ti2—O23	−18.54 (11)
Ti2—C24—C25—C21	−64.9 (3)	Ti3—O32—Ti2—C23	−130.9 (3)
C23—C24—C25—C30	−179.2 (4)	Ti3—O32—Ti2—C24	−105.40 (14)
C29—C24—C25—C30	0.2 (6)	Ti3—O32—Ti2—C25	−128.46 (12)
Ti2—C24—C25—C30	116.3 (4)	Ti3—O32—Ti2—C21	−162.95 (11)
C23—C24—C25—Ti2	64.5 (3)	Ti3—O32—Ti2—C22	−177.08 (13)
C29—C24—C25—Ti2	−116.1 (4)	Ti3—O32—Ti2—Ti1	64.97 (8)
C22—C21—C25—C24	−0.4 (4)	Ti1—O13—Ti3—O23	−48.83 (17)
C26—C21—C25—C24	−179.5 (4)	Ti1—O13—Ti3—O32	37.88 (18)
Ti2—C21—C25—C24	64.4 (3)	Ti1—O13—Ti3—C33	−159.7 (2)
C22—C21—C25—C30	178.5 (4)	Ti1—O13—Ti3—C34	−151.15 (15)
C26—C21—C25—C30	−0.6 (6)	Ti1—O13—Ti3—C31	149.06 (17)
Ti2—C21—C25—C30	−116.7 (4)	Ti1—O13—Ti3—C32	144.55 (17)
C22—C21—C25—Ti2	−64.8 (2)	Ti1—O13—Ti3—C35	−178.96 (17)
C26—C21—C25—Ti2	116.1 (4)	Ti1—O13—Ti3—Ti2	−5.44 (15)
C35—C31—C32—C33	0.9 (4)	Ti2—O23—Ti3—O13	83.29 (11)
C36—C31—C32—C33	177.3 (4)	Ti2—O23—Ti3—O32	−18.55 (10)
Ti3—C31—C32—C33	−64.2 (3)	Ti2—O23—Ti3—C33	−128.60 (12)
C35—C31—C32—C37	−179.1 (4)	Ti2—O23—Ti3—C34	−163.27 (11)
C36—C31—C32—C37	−2.6 (7)	Ti2—O23—Ti3—C31	−132.4 (3)
Ti3—C31—C32—C37	115.8 (4)	Ti2—O23—Ti3—C32	−106.94 (14)
C35—C31—C32—Ti3	65.2 (3)	Ti2—O23—Ti3—C35	−177.19 (14)
C36—C31—C32—Ti3	−118.4 (4)	Ti2—O23—Ti3—Ti1	62.48 (8)
C31—C32—C33—C34	−0.5 (4)	Ti2—O32—Ti3—O13	−81.55 (12)
C37—C32—C33—C34	179.5 (4)	Ti2—O32—Ti3—O23	18.50 (10)
Ti3—C32—C33—C34	−64.8 (2)	Ti2—O32—Ti3—C33	108.93 (14)
C31—C32—C33—C38	−179.9 (4)	Ti2—O32—Ti3—C34	113.2 (2)
C37—C32—C33—C38	0.1 (6)	Ti2—O32—Ti3—C31	169.28 (14)
Ti3—C32—C33—C38	115.8 (4)	Ti2—O32—Ti3—C32	138.16 (15)
C31—C32—C33—Ti3	64.3 (3)	Ti2—O32—Ti3—C35	171.69 (17)
C37—C32—C33—Ti3	−115.7 (4)	Ti2—O32—Ti3—Ti1	−64.63 (8)
C32—C33—C34—C35	−0.1 (4)		

Symmetry code: (i) −*x*+1, *y*, −*z*+1/2.
