# Supplementary figures and images for: Crystal structure of μ-oxido-1,1′κ2 O:O-bis{tetra-μ-oxido-1:2κ2 O:O;1:3κ2 O:O;2:3κ4 O:O-tris[1,2,3(η5)-penta­methyl­cyclo­penta­dien­yl]-trianglo-trititanium(IV)}
Source: Acta Crystallogr E Crystallogr Commun. 2015 Mar 21;71(Pt 4):m97. doi: 10.1107/S2056989015004041 (PMC4438812; doi:10.1107/S2056989015004041)

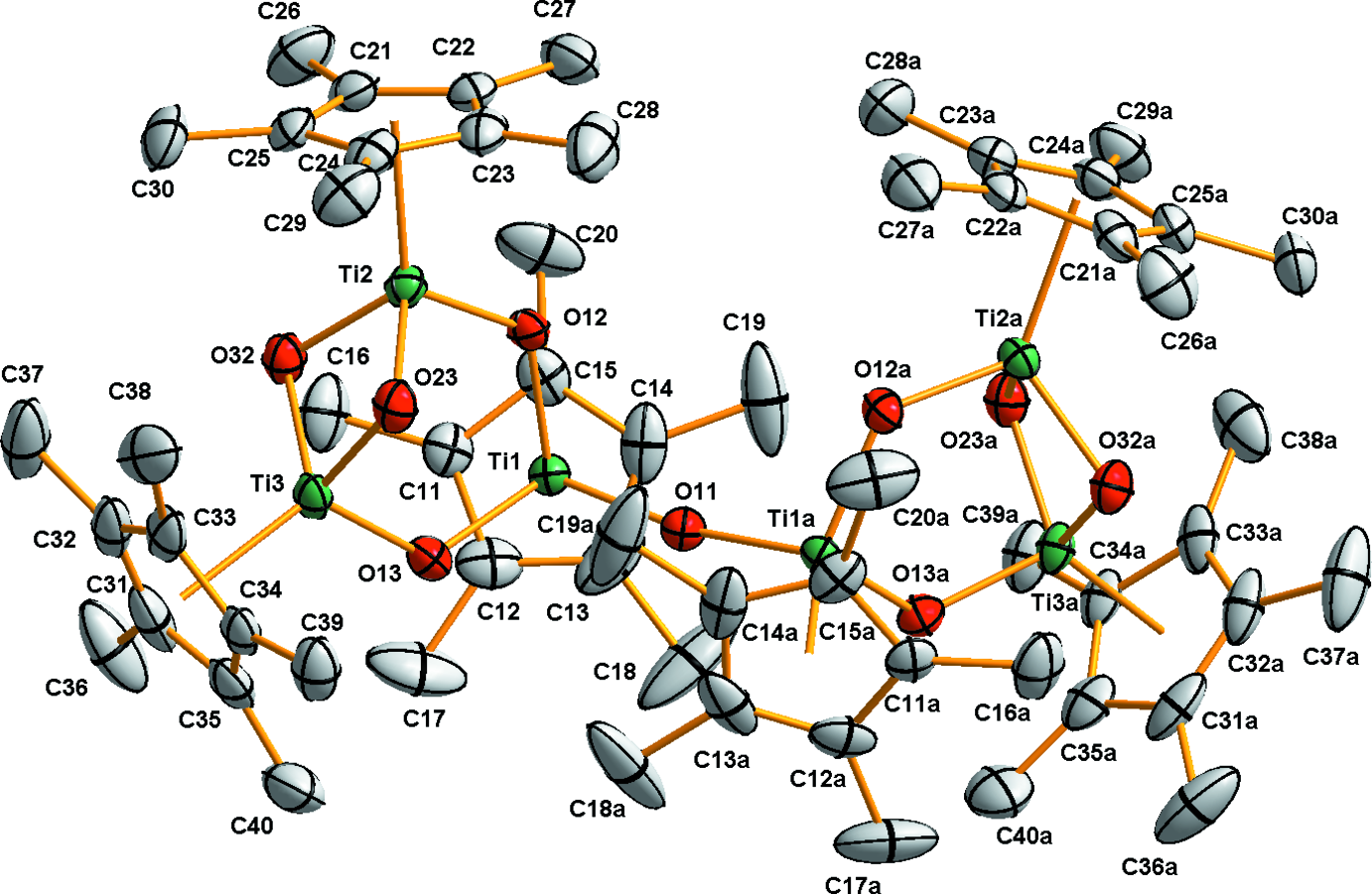

Supplement: Supplementary file 4 [file e-71-00m97-fig1.tif]
